# Previous infection with *Staphylococcus aureus* strains attenuated experimental encephalomyelitis

**DOI:** 10.1186/1471-2202-15-8

**Published:** 2014-01-09

**Authors:** Thais Graziela Donegá França, Fernanda Chiuso-Minicucci, Sofia Fernanda Gonçalves Zorzella-Pezavento, Larissa Lumi Watanabe Ishikawa, Larissa Camargo da Rosa, Priscila Maria Colavite, Camila Marques, Maura Rosane Valério Ikoma, Maria de Lourdes Ribeiro de Souza da Cunha, Alexandrina Sartori

**Affiliations:** 1 Department of Microbiology and Immunology, Biosciences Institute, Univ Estadual Paulista (UNESP), Distrito de Rubião Júnior s/n, 18618-000 Botucatu, São Paulo, Brazil; 2 Laboratório de Citometria de Fluxo, Fundação Dr. Amaral Carvalho, Jaú, São Paulo, Brazil

**Keywords:** *S. aureus*, Experimental autoimmune encephalomyelitis, Toxic shock syndrome toxin 1

## Abstract

**Background:**

Bacterial superantigens are potent T cell activators that can activate T cells with specificity for antigens of the central nervous system (CNS). In this study, we compared the effect of two *S. aureus* strains on experimental autoimmune encephalomyelitis (EAE) development. C57BL/6 female mice were infected with *S. aureus* ATCC 51650, which produces toxic shock syndrome toxin 1 (TSST-1+) or *S. aureus* ATCC 43300, which does not produce toxins (TOX-). Three days later, the animals were subjected to EAE induction by immunization with myelin oligodendrocyte glycoprotein (MOG). The weight variation, disease incidence and clinical score were recorded daily. Cytokines and Foxp3+ regulatory T cells in the brain were evaluated during the acute disease phase. Cytokines and Foxp3+ regulatory T cells in the spleen and histopathological analysis of the CNS were assessed during the chronic stage.

**Results:**

Previous infection with both strains similarly decreased the clinical score; however, only the TSST-1+ strain clearly diminished inflammation in the CNS. The infections also modulated cytokine production in the spleen and CNS. Reduced production of IL-5 and IL-10 was detected in MOG-stimulated spleen cultures in the TOX- and TSST-1+ infected groups, respectively. In *S. aureus* stimulated cultures, there was an increased production of IFN-γ and IL-10 in both infected groups and an increased level of IL-5 in the TSST-1+ group. CNS infiltrating cell cultures from previously infected mice produced less IL-17 in response to MOG and more IFN-γ in response to *S. aureus* stimulation.

**Conclusions:**

These results indicated that both strains attenuated clinical EAE manifestations, but only TSST-1 clearly decreased CNS inflammation.

## Background

Multiple sclerosis (MS) is a demyelinating disease of the central nervous system (CNS), which is mainly mediated by T cells that are specific for the myelin self-antigen. These autoreactive T cells are generated in the periphery and cross the blood-brain barrier to the brain parenchyma where they initiate an autoimmune attack on the myelin sheath [1,2]. MS has been histopathologically characterized by four main findings: inflammation, demyelination, axonal damage and gliosis. Much of the knowledge about MS is derived from studies on experimental autoimmune encephalomyelitis (EAE). This disease can be induced in a variety of animals, particularly in rodents, providing models of acute monophasic, relapsing remitting and chronic progressive CNS inflammation [2]. Although many gaps still exist in the understanding of MS immunopathogenesis, it is widely believed that this complex pathology involves both host genetic and environmental factors [1]. Infectious disease agents can modulate autoimmune diseases in many different ways, such as triggering these pathologies or, contrarily, preventing their development [3,4]. *S. aureus* is one of the most prevalent pathogens in the human population; colonizing the anterior nares of 20-60% of the population. These Gram-positive bacteria can cause superficial skin infections, such as abscesses and impetigo, or serious invasive infections such as septic arthritis, osteomyelitis, endocarditis [5-7] and food poisoning [8]. Most *S. aureus* strains secrete an array of extracellular enzymes, which facilitate tissue destruction and spreading and membrane damaging toxins that cause cytolytic effects on host cells and tissue damage [9,10]. Currently, 20 serologically distinct staphylococcal superantigens (SAgs) have been described, including TSST-1 and the enterotoxins A-E and G-J [11]. *S. aureus* SAgs can activate a high proportion of T cells due to their ability to bind to both MHC class II molecules in antigen presenting cells and specific V-β regions in the T cell receptor [1,12]. This activation results in the polyclonal stimulation of T cells and an elevated production of proinflammatory cytokines [1,13]. Experimental and epidemiological evidence support the theory that *S. aureus* that produces SAgs may be implicated in the genesis of MS [1,14,15]. In this context, the main objective of this investigation was to compare the effects of two *S. aureus* strains, one strain that produces TSST-1 and an other strain that does not produce any SAgs, on EAE development.

## Results

### Clinical disease severity is reduced by a previous *S. aureus* infection

**Figure 1:**
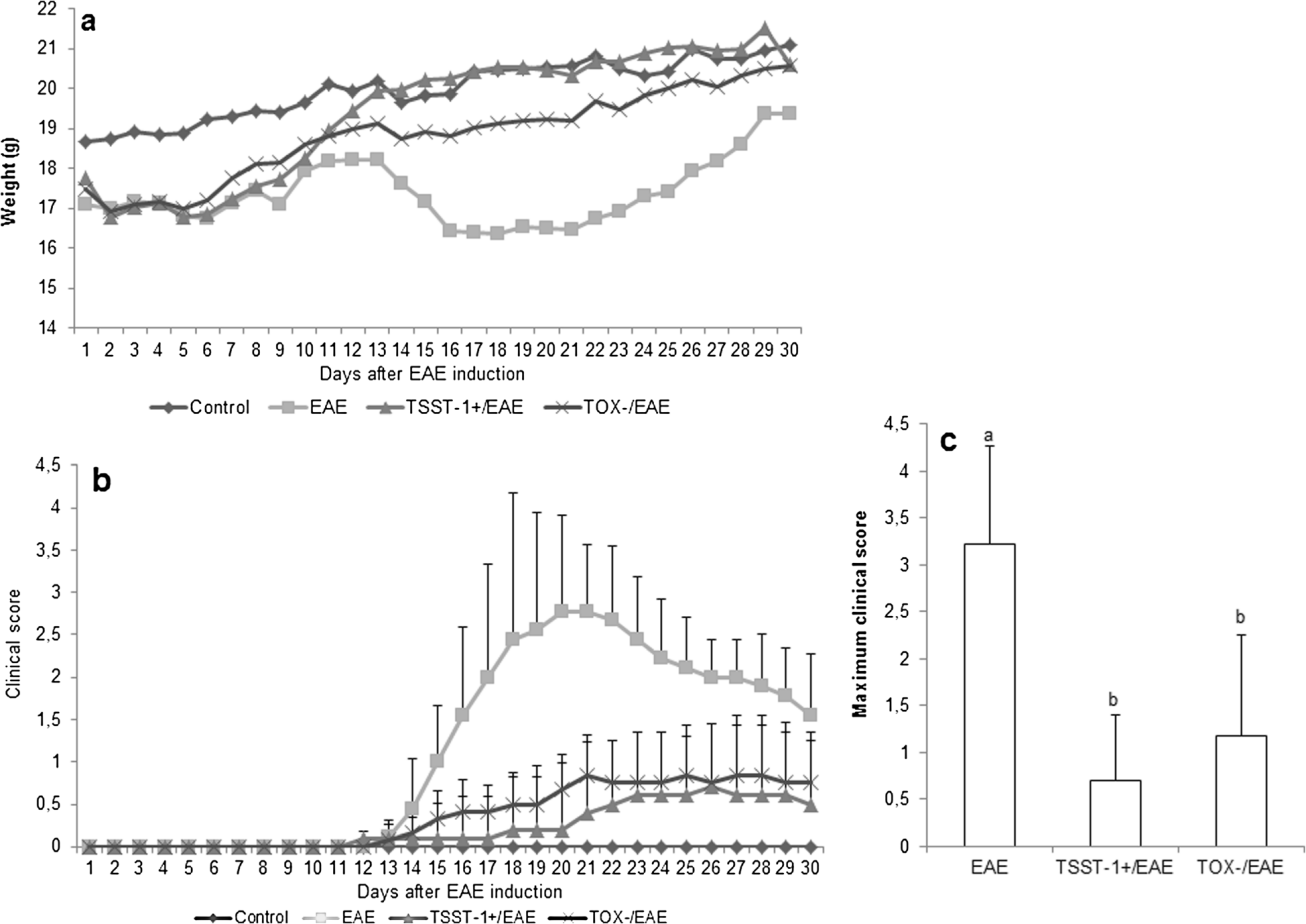
**Effect of previous infection with *S. aureus *in body weight and clinical score.** C57BL/6 mice were infected with *S. aureus* TSST-1+ and TOX-. Three days later, the animals were subjected to EAE induction, and their body weight and clinical scores were evaluated daily. The profile of the animals’ weight during 30 days **(a)**, clinical score **(b)** and maximum clinical score **(c)**. Data are presented as the mean ± SE of 9-12 mice and are representative of two independent experiments, p < 0.05. The statistical analysis (ANOVA or ANOVA on Ranks with Dunn’s method) indicates significant differences between EAE and both infected groups. These differences began on day 15 and remained significant until day 30. No differences were observed between the two infected groups.

The EAE control group (mice immunized with myelin oligodendrocyte glycoprotein (MOG) without a previous *S. aureus* infection) presented the expected clinical alterations. These animals showed a reduction in body weight (Figure 1a), which coincided with the highest clinical scores observed during the acute phase of the disease (Figure 1b). Previous infection with both *S. aureus* strains prevented this reduction in body weight (Figure 1a) and also decreased the clinical scores (Figure 1b and 1c). This protective effect on disease severity was also detected when the disease incidence was examined. As shown in Table 1, 89% of the animals from the EAE control group were ill whereas the incidence in the TOX-/EAE and TSST-1+/EAE groups was 67% and 36%, respectively.

**Table 1:** **Effect of previous infection with *S. aureus *** **on EAE development (%)**

	**Number of sick animals**	**Percentage of sick animals**	**P**
EAE (n = 9)	8/9	89	p = 0.051
TSST-1^+^/EAE (n = 10)	4/10	36	
TOX^-^/EAE (n = 12)	8/12	67	

### Inflammation intensity in the CNS is reduced by previous infection with *S. aureus* TSST-1

**Figure 2:**
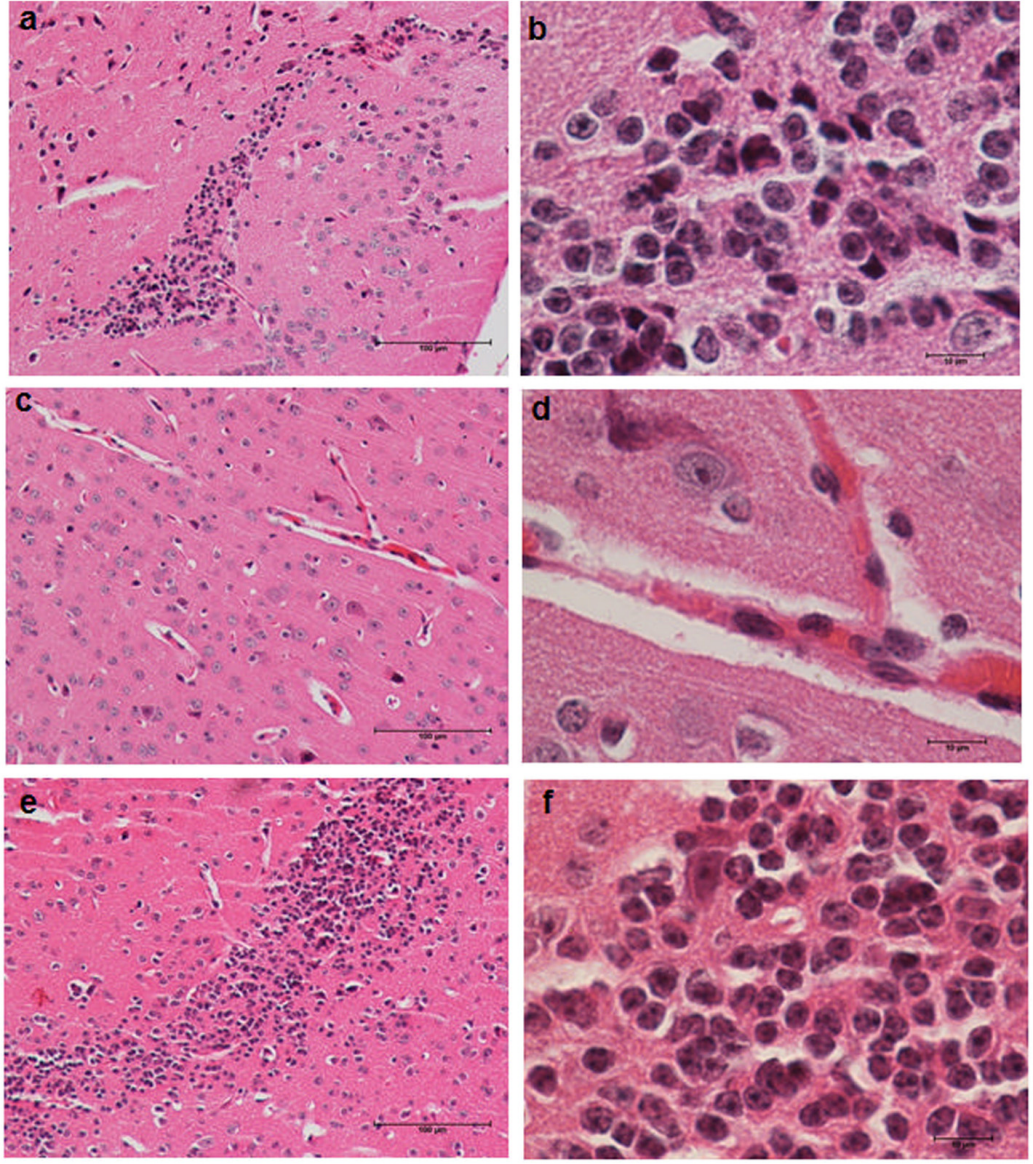
**Inflammation in the brain of mice with EAE: effect of previous infection with *S. aureus*.** C57BL/6 mice were infected with *S. aureus* TSST-1+ and TOX-, and 3 days later, they were subjected to EAE induction. Brain inflammatory infiltrates in mice with EAE **(a, b)**, mice previously infected with *S. aureus* TSST-1 strain subjected to EAE **(c, d)** and mice previously infected with *S. aureus* TOX- strain subjected to EAE **(e, f)** were evaluated 30 days after disease induction. The panel is representative of 6-7 animals/group. Magnification is 10 x and 100 x in the left and right panel, respectively.

**Figure 3:**
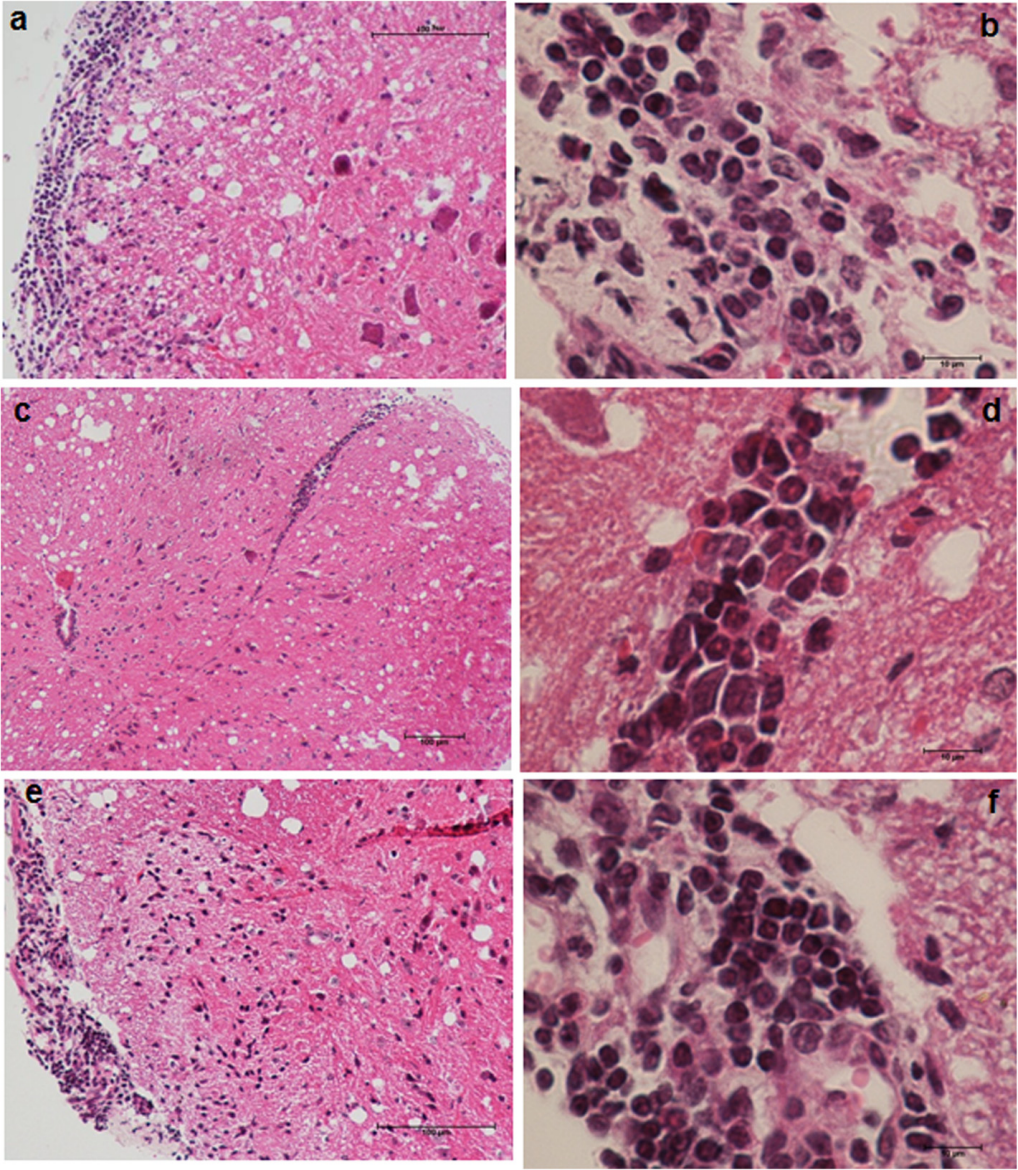
**Inflammation in the spinal cord of mice with EAE: effect of previous infection with *S. aureus*.** C57BL/6 mice were infected with *S. aureus* TSST-1+ and TOX-, and 3 days later, they were subjected to EAE induction. The lumbar spinal cord inflammatory infiltrates in mice with EAE **(a, b)**, mice previously infected with *S. aureus* TSST-1 strain subjected to EAE **(c, d)** and mice previously infected with *S. aureus* TOX- strain subjected to EAE **(e, f)** were evaluated 30 days after disease induction. The panel is representative of 6-7 animals/group. Magnification is 10 x and 100 x in the left and right panel, respectively.

Typical lesions characterized by intense inflammatory infiltrates were observed in the brains (Figure 2a) and lumbar spinal cords (Figure 3a) in the animals in the EAE control group. Comparable findings were observed in animals that were infected with the TOX- strain prior to EAE induction (Figures 2e and 3e). In contrast, the group that was previously infected with the TSST-1+ strain presented a very discrete inflammatory infiltration, which was restricted to the perivascular region in the brain and lumbar spinal cord (Figures 2c and 3c, respectively).

### Peripheral cytokine production is modulated by *S. aureus* infections

**Figure 4:**
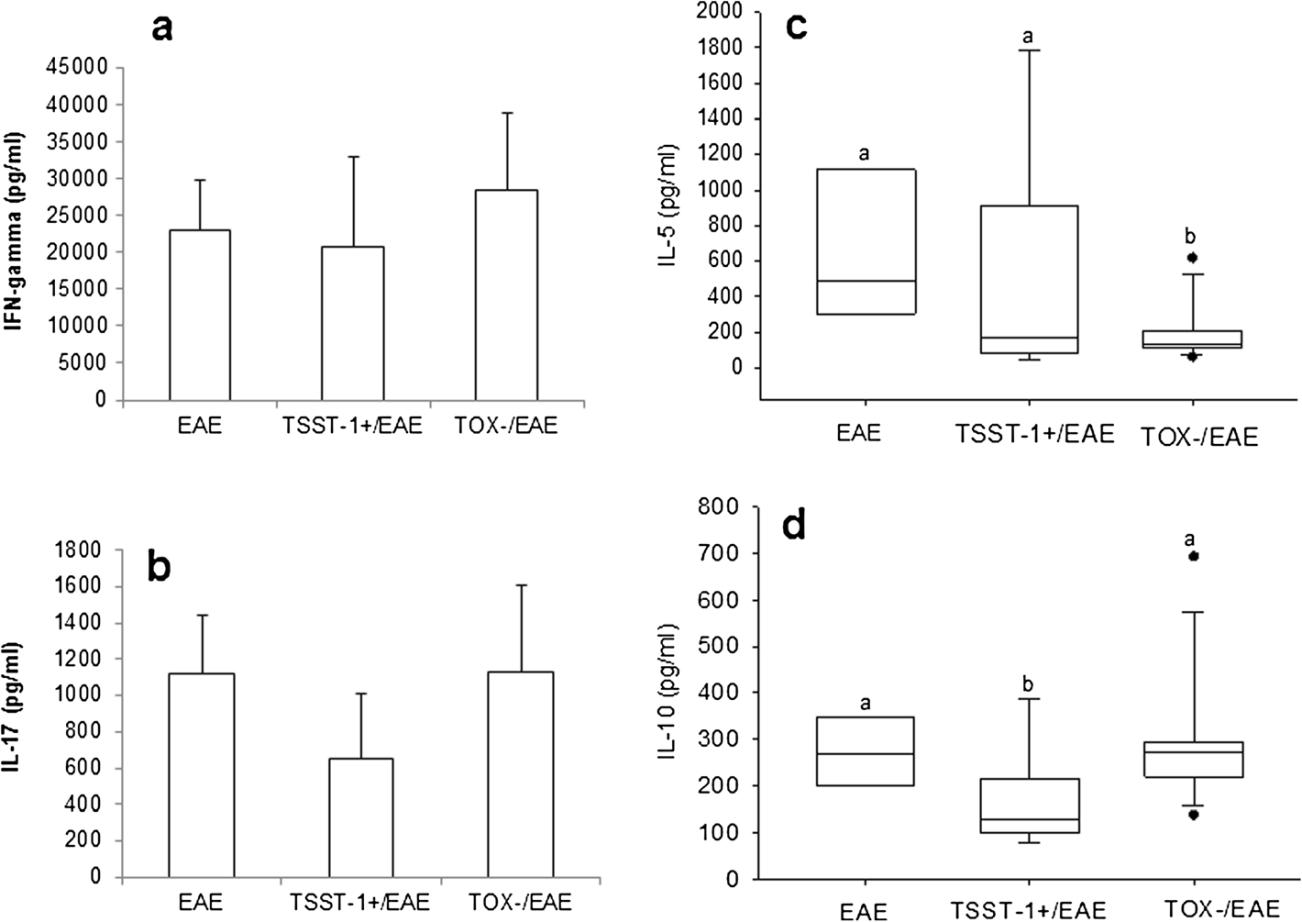
**Production of cytokines by spleen cells stimulated with MOG.** C57BL/6 mice were infected with *S. aureus* strains, and 3 days later, they were subjected to EAE induction. Cytokine production was tested 30 days after EAE induction. IFN-γ **(a)**, IL-17 **(b)**, IL-5 **(c)** and IL-10 **(d)** production were assayed in spleen cell cultures that were re-stimulated *in vitro* with MOG. Data are expressed as the mean ± SE of 5-12 mice. Different letters represent significant differences between the groups. The statistical analysis (ANOVA or ANOVA on Ranks with Dunn’s method) indicate significant differences between EAE and both infected groups, p<0.05.

**Figure 5:**
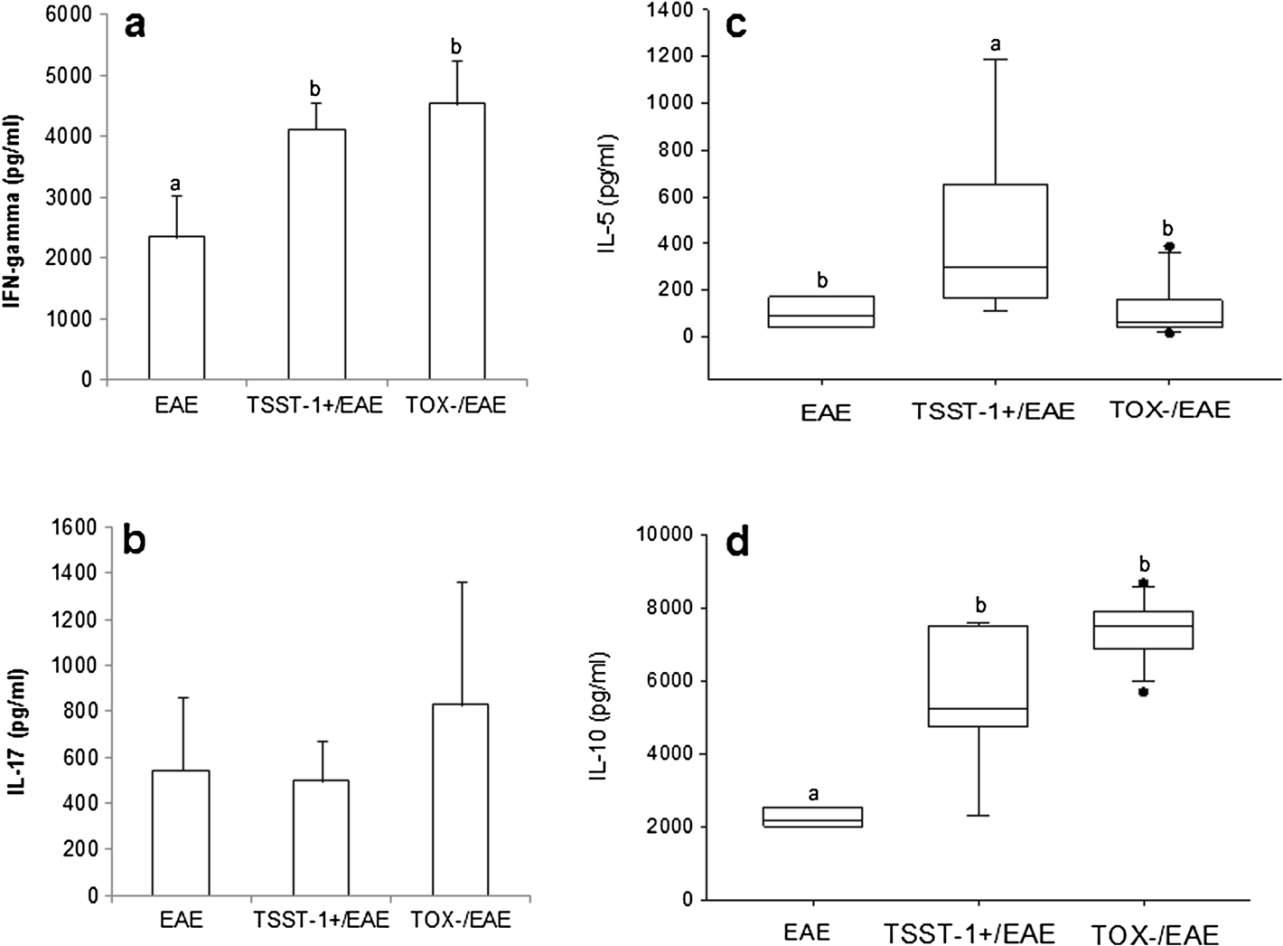
**Production of cytokines by spleen cells stimulated with SAC*.*** C57BL/6 mice were infected with *S. aureus* strains, and 3 days later, they were subjected to EAE induction. Cytokine production was tested 30 days after EAE induction. IFN-γ **(a)**, IL-17 **(b)**, IL-5 **(c)** and IL-10 **(d)** production were assayed in spleen cell cultures that were re-stimulated *in vitro* with SAC. Data are expressed as the mean ± SE of 5-12 mice. Different letters represent significant differences between the groups. The statistical analysis (ANOVA or ANOVA on Ranks with Dunn’s method) indicate significant differences between EAE and both infected groups, p<0.05.

Production of IFN-γ and IL-17 induced by MOG was not affected by previous infection with either strain of *S. aureus*, as illustrated in Figure 4a and 4b, respectively. Significant reductions in IL-5 (Figure 4c) and IL-10 (Figure 4d) were observed in the groups that were previously infected with TOX- and TSST-1+, respectively. Some clear differences were observed in the levels of these cytokines when splenic cultures were stimulated with *S. aureus* antigen (SAC). IFN-γ and IL-10 production was significantly elevated in the previously infected group compared to the EAE group (Figure 5a and d, respectively). IL-5 production by the TSST-1+ group was also significantly higher compared to the EAE and TOX-/EAE groups (Figure 5c). Similar levels of IL-17 were found in the three experimental groups (Figure 5b).

### In situ (CNS) cytokine production is modulated by previous *S. aureus* infections

**Figure 6:**
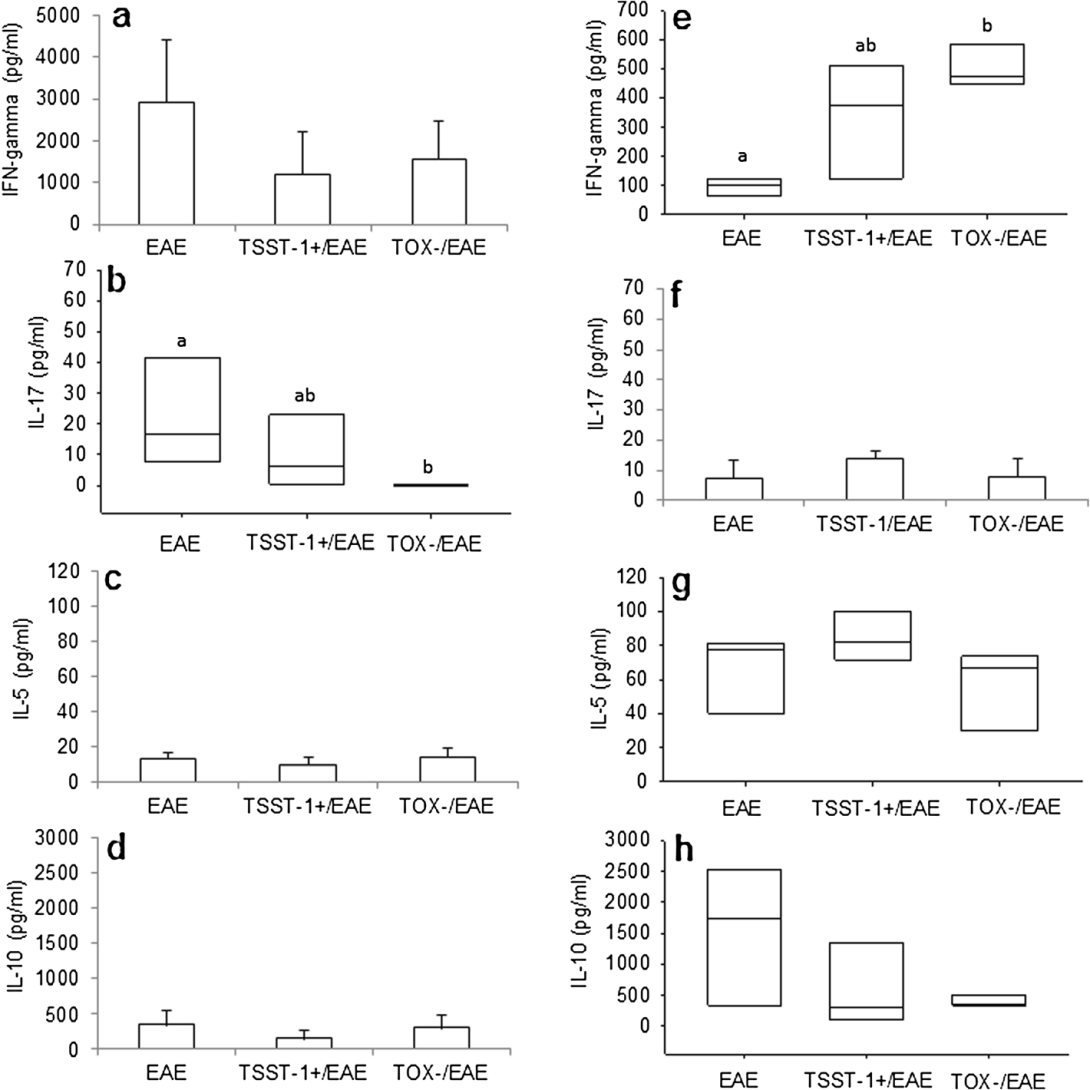
**Production of cytokines by CNS infiltrating cells stimulated with MOG or SAC.** C57BL/6 mice were infected with *S. aureus* strains, and 3 days later, they were subjected to EAE induction. Cytokine production was tested 19 days after EAE induction (acute phase) in CNS cell cultures. IFN-γ **(a)**, IL-17 **(b)**, IL-5 **(c)** and IL-10 **(d)** production were assayed in cultures that were re-stimulated *in vitro* with MOG. IFN-γ **(e)**, IL-17 **(f)**, IL-5 **(g)** and IL-10 **(h)** production were assayed in cultures that were re-stimulated *in vitro* with SAC. Data are expressed as the mean ± SE of 5-12 mice. Different letters represent significant differences between groups. Statistical analysis (ANOVA or ANOVA on Ranks with Dunn’s method) indicates significant differences between EAE and both infected groups, p< 0.05.

CNS infiltrating cells from the EAE, TSST-1+/EAE and TOX-/EAE groups were cultured in the presence of MOG or SAC. In MOG-stimulated cultures, the production of IFN-γ, IL-5 and IL-10 was similar between the 3 groups (Figure 6a, c and d). However, previously infected animals produced less IL-17 than the animals in the EAE control group (Figure 6b). In the SAC-stimulated cultures, IFN-γ levels were higher in previously infected mice compared to the mice in the EAE control group. No differences were observed in the IL-17, IL-5 and IL-10 production in these cultures. These results are shown in Figure 6f, g and h, respectively.

### Previous infection with TOX- *S. aureus* down-modulated the percentage of Foxp3+ regulatory T cells in the CNS

**Figure 7:**
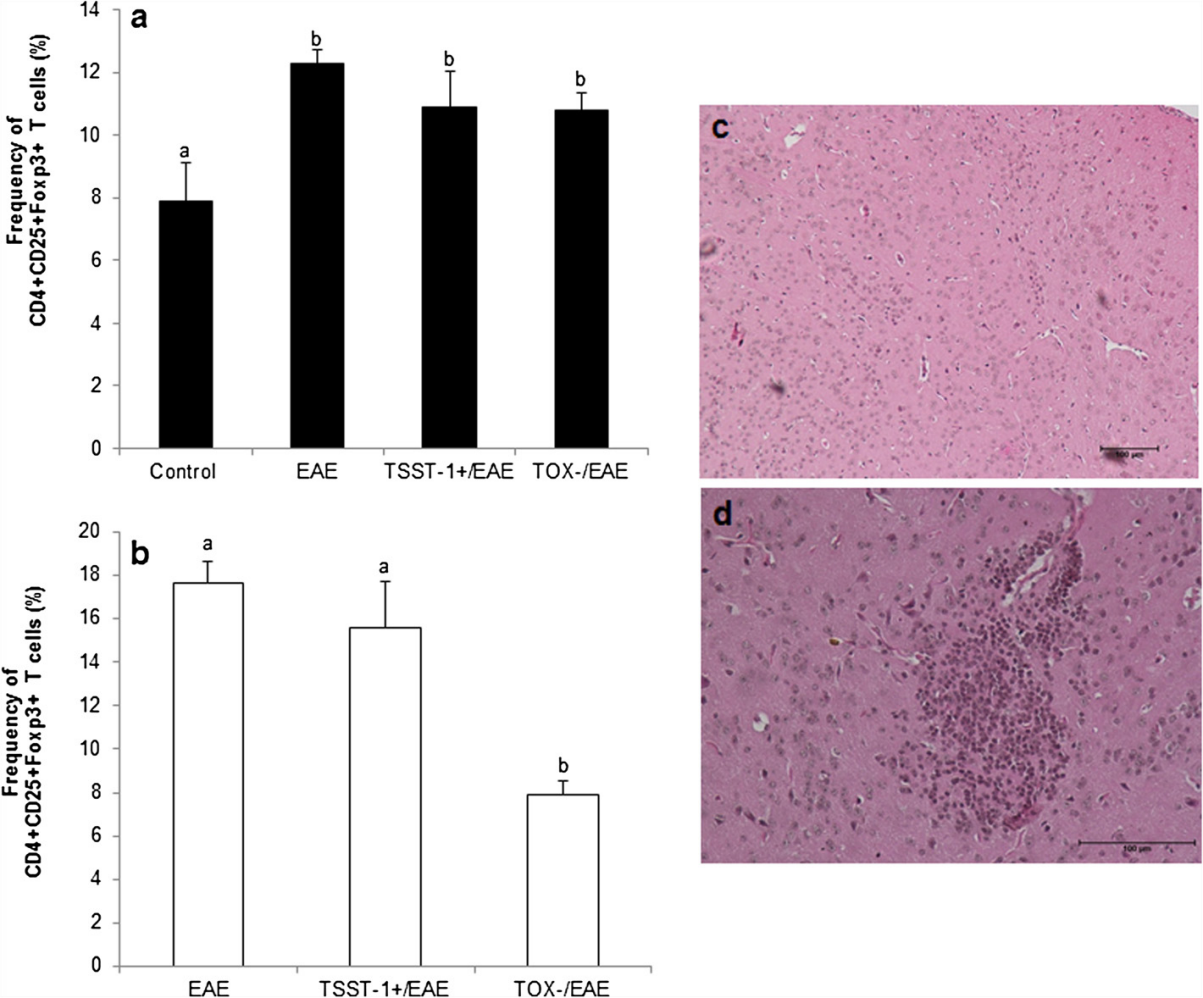
**Frequency of regulatory T cells in the spleen and CNS of mice with EAE: effect of previous infections with *S. aureus.*** The percentage of CD4 + CD25 + Foxp3+ T cells was evaluated in the spleen **(a)** and CNS **(b)**. Brain inflammatory infiltrates in mice previously infected with *S. aureus* TSST-1 **(c)** or with *S. aureus* TOX- strain **(d)** were evaluated 15 days after the infection. Data were expressed as the mean ± SE of 6-9 mice in Figures 7a and b. Different letters represent significant differences between groups. The statistical analysis (ANOVA with Dunn’s or Holm-Sidak method) indicates significant differences between EAE and both infected groups, p<0.05. Micrographs **(c** and **d)** are representative of 5-6 animals/group.

Animals with EAE presented a significant increase in the frequency of splenic CD4+ CD25+ Foxp3+ T cells compared to the negative control group (Figure 7a). Infections with TSST-1+ and TOX- strains before EAE induction did not alter the percentage of these T cells in this peripheral lymphoid organ (Figure 7a). The presence of these regulatory cells was concomitantly evaluated in the CNS. As shown in Figure 7b, the number of CD4+ CD25+ Foxp3+ T cells was similar in the EAE and TSST-1+ groups, but was significantly lower in the group that was previously infected with the TOX- strain. Histopathological analysis indicated that the brains from the mice that were solely infected with TOX- and were not subjected to EAE induction presented abundant inflammatory infiltrates 15 days after infection. In contrast to this result, mice infected with the TSST-1+ strain presented no inflammation in the corresponding time period (Figure 7d and c, respectively).

## Discussion

Multiple sclerosis (MS) is a demyelinating disease of the central nervous system (CNS) that is characterized by an autoimmune inflammatory process involving myelin antigens [2]. Experimental autoimmune encephalomyelitis is currently the most widely accepted animal model to investigate this pathology. Epidemiological studies have identified several environmental risk factors that contribute to the development of this disease, such as viral infections, smoking and depleted vitamin D serum levels [16]. Although SAgs have been implicated in the pathogenesis of different autoimmune diseases, such as type I diabetes, Kawasaki disease and MS [17], their effects have not been systematically tested in these pathologies. In the present investigation, we evaluated the effect of previous infections with *S. aureus* on EAE development. To achieve this, female C57BL/6 mice were infected with 2 distinct *S. aureus* strains; one strain was a producer of the TSST-1 toxin (ATCC 51650), and the other strain did not produce any SAgs (ATCC 43300). Three days after infection, the animals were subjected to EAE induction, and the disease evolution was compared with a control group that had not been infected before EAE induction. Previous inoculation with both strains clearly induced a protective effect that was characterized by the appearance of a much more benign disease. Previously infected animals did not lose weight during the acute phase and also presented a less severe sickness with lower clinical scores. In addition to these less serious clinical manifestations, there was also a lower disease incidence. A comparison of all of these parameters indicated that the TSST-1+ strain was more protective than the TOX- strain. In addition, the first signals of paralysis in the TSST-1+ infected group were delayed. In this group, these clinical symptoms appeared only on the 21^st^ day after EAE induction, compared to the EAE control group, which exhibited symptoms by the 14^th^ day. To examine the mechanisms by which these two *S. aureus* strains protected against EAE development, cytokine production by the spleen and CNS infiltrating cell cultures were concomitantly evaluated. The similar IFN-γ and IL-17 levels in the MOG-stimulated spleen cultures from the three experimental groups suggest that previous infection is not down-regulating the peripheral production of encephalytogenic cytokines. However, a significant down-regulation of IL-5 and IL-10 was observed in the TOX-/EAE and TSST-1+/EAE-infected animals, respectively. In this context, we hypothesized that this decreased production of anti-inflammatory cytokines was due to a migration of specific T cell clones from the periphery to the CNS. However, an evaluation of IL-5 and IL-10 production by cells infiltrating the CNS was not consistent with this interpretation. Interestingly, there was a lower production of IL-17 in the CNS of mice that were previously infected with *S. aureus* compared to the positive control animals, i.e., the EAE group. We believe that this is an important finding because IL-17 is described as a relevant mediator of EAE and MS immunopathogenesis [18,19]. Because the peripheral production of IL-17 stimulated by MOG was similar between the three groups, this lower local level of IL-17 could be due to a decreased migration of Th17-specific clones to the CNS or to a local down-modulation of IL-17 production. To test the last possibility, we analyzed the cytokine production specifically induced by *S. aureus* (SAC) in the periphery (spleen) and in the CNS. Previous infection was associated with an accentuated immune response characterized by a significant production of IFN-γ, IL-5 and IL-10. SAC-specific clones capable of producing anti-inflammatory cytokines could migrate to the CNS and down-modulate IL-17 production. A theoretical basis for this possibility was found in the literature. Initially described as a product of Th2 cells, IL-10 is now recognized as being secreted by nearly every cell type of the immune system and is able to inhibit the inflammatory process [20]. In the context of MS and EAE, IL-10 clearly elicits beneficial effects on these diseases. In MS patients, for example, IL-10 levels are increased in the serum during disease remission [21]. In addition, the efficacy of IFN-β and glatiramer acetate, two widely employed MS treatments, is partially attributed to the induction of IL-10 [21-23]. Genetic studies using the EAE model also showed that IL-10 deletion enhanced EAE disease severity while over-expression of this cytokine protected mice [24]. Moreover, treatment of EAE with a herpes simplex virus type 1 vector expressing IL-5 ameliorated EAE and decreased the number of infiltrating lymphocytes in the brain [25]. Similarly, treatment of experimental autoimmune neuritis with recombinant IL-5 markedly reduced clinical paralysis, weight loss, demyelination as well as infiltration of Th1, Th17 and Tc cells and macrophages in nerves [26]. Despite the significant peripheral production of IL-10 in mice infected with both strains and IL-5 in mice infected with the TOX- strain, the levels of these cytokines were statistically similar in the CNS of the three experimental groups. Taken together, the cytokine results suggest that down-modulation of IL-17 in the brain was not mediated by IL-10 or IL-5 production in the CNS. Considering that the balance between Th17 and regulatory T cells is critical in autoimmune diseases [27] and that a high frequency of regulatory T cells migrate to the CNS during EAE recovery [28,29], we analyzed the frequency of Foxp3+ regulatory T cells in the spleen and at the site of inflammation. The proportion of CD4+ CD25+ Foxp3+ cells in the spleen and CNS was significantly elevated in mice with EAE, as shown in this study and previous studies [18,28]. The supposition that the protection by previous *S. aureus* infection was mediated by an increased number of Foxp3+ Treg cells was not confirmed. Previous infections did not increase the number of Foxp3+ cells in either the spleen or the CNS. In contrast with our expectations, the number of CD4+ CD25+ Foxp3+ cells was significantly decreased in the TOX-/EAE group. This finding was consistent with the presence of a strong inflammatory process in the CNS of this group, although the animals were clearly protected from the disease. The histopathological analysis of the infected animals that were not subjected to EAE induction showed that infection with the TOX- strain, but not with the TSST-1strain, triggered a clear inflammatory process in the brain. Taken together, these results suggest that the TOX- strain or its secreted components can cross the blood-brain barrier and elicit a local accumulation of inflammatory cells. Penetration of *S. aureus* across the blood-brain barrier has recently been described [30] and provides support for this idea. In this scenario, the lower number of Foxp3+ cells in TOX-infected animals could be due to a “dilutive effect” caused by the infiltration of phagocytic cells that are trying to eliminate a local *S. aureus* infection. Our initial hypothesis that the TSST-1 strain could increase disease severity due to its superantigenicity was not confirmed by our findings. Considering the various parameters (lower clinical score, lower disease incidence and very discrete inflammation in the CNS), we believe that this strain was more protective than the TOX- strain. Thus, we hypothesize that these two *S. aureus* strains are protecting the animals from EAE development using different molecular mechanisms or more than one immunomodulatory via. In this scenario, we could imagine that the TSST-1 strain was more effective because it employed a stronger immunoregulatory mechanism or multiple mechanisms. In this sense, it has been demonstrated that the TSST-1 toxin was able to cause apoptosis of myelin T cell clones [31]. However, we could not make a direct comparison of these results with the literature because there were no experimental approaches similar to the one employed by our study. Thus, this is the first direct demonstration that an *S. aureus* infection was able to decrease the severity of EAE. Further studies are necessary to highlight the protective mechanisms that are triggered by *S. aureus* infection to protect against EAE development. It will be relevant to establish if these two strains present differences related to dissemination in the CNS as well as in the secretion of extracellular adherence protein (Eap). This protein is endowed with the ability to prevent EAE development by inhibiting infiltration of inflammatory cells into the CNS [32].

## Conclusion

These findings indicate that both TSST-1+ and TOX- *S. aureus* strains were able to attenuate EAE severity. They also suggest that, in contrast to our expectations, the TSST-1+ strain was more protective than the TOX- strain.

## Methods

### Experimental design

Mice were infected with *S. aureus* strains. Three days later, they were subjected to EAE induction by immunization with MOG, which was emulsified with complete Freund’s adjuvant. The effect of the infections on EAE development was evaluated by a clinical follow-up (weight variation and clinical score) and also by histopathological analysis of the CNS. The potential immunoregulatory effect of the infections was confirmed by cytokine production and quantification of Foxp3+ regulatory T cell. Cell cultures were stimulated with MOG and *S. aureus* Cowan I (SAC). Cytokines and Foxp3+ regulatory T cells were evaluated in the CNS during the acute disease phase, whereas cytokine production by the spleen and brain and lumbar spinal cord histopathological analysis were performed during the chronic phase. An additional histopathological analysis was performed in the brains of the mice that were solely infected (15 days of infection).

### Animals

C57BL/6 female mice (8-10 weeks old) were purchased from CEMIB (UNICAMP, São Paulo, SP, Brazil). The animals were provided with sterilized food and water *ad libitum*. The mice were manipulated according to the ethical guidelines adopted by the Brazilian College of Animal Experimentation. All of the experimental protocols were approved by the local Ethics Committee (Ethics Committee for Animal Experimentation, Bioscience Institute, Univ. Estadual Paulista).

### Bacterial strains: growth conditions and mice infection

Two *Staphylococcus aureus* strains obtained from the American Type Culture Collection (ATCC) were used in this study. The ATCC 51650 strain was characterized by TSST-1 production (TSST-1+), while the ATCC 43300 did not produce any toxin (TOX-). These strains were initially cultured in blood agar and incubated at 37°C for 24 h. Isolated colonies were inoculated into brain heart broth (BHI, Merck) and incubated at 37°C for 24 h. The bacteria were collected by centrifugation, washed three times and resuspended in cold sterile saline as previously described by França et al., 2009 [33]. The animals were intraperitoneally injected with 300 μL of the bacterial suspensions. The bacterial load in the blood was confirmed 3 days after infection and was found to be similar for both strains.

### EAE induction

MOG_35-55_ peptide (MEVGWYRSPFSRVVHLYRNGK) was synthesized by Proteimax, São Paulo, Brazil. EAE was induced as previously described (Zorzella-Pezavento et al., 2013). Briefly, the mice were immunized subcutaneously with 150 μg of MOG35–55 peptide emulsified in CFA containing 400 μg of BCG. The mice also received 2 doses of 200 ng of *Bordetella pertussis* toxin (Sigma) intraperitoneally 0 and 48 h after immunization. The animals were examined daily and the disease intensity was graded as: 0 - no disease, 1 - limp tail, 2 - weak/partially paralyzed hind legs, 3 - completely paralyzed hind legs, 4 - complete hind and partial front leg paralysis, 5 - complete paralysis/death.

### CNS-Infiltrating Cell Isolation

The mice were anesthetized with ketamine/xylazine and perfused with 10 mL of saline solution. The brain and spinal cord were excised, macerated, and maintained in 4 mL of RPMI (Sigma) supplemented with 2.5% collagenase D (Roche) at 37°C in a 5% CO_2_ incubator. Forty-five min later, the suspensions were washed in RPMI and centrifuged at 450 × g for 15 min at 4°C. The cells were resuspended in 37% percoll (GE Healthcare) and gently laid over 70% percoll in tubes of 15 mL. The tubes were centrifuged at 950 × g for 20 min with the centrifuge breaks turned off. After centrifugation, the ring containing the mononuclear cells was collected, washed in RPMI, and centrifuged at 450 × g for 5 min. The cellular suspensions were then resuspended in complete RPMI medium, quantified, and analyzed.

### Cell culture conditions and cytokine assays

CNS-infiltrating cells and spleen cells were adjusted to 2 × 10^5^ cells/mL and 5 × 10^6^ cells/mL, respectively, in RPMI medium supplemented with 5% fetal calf serum, 20 mM glutamine and 40 IU/mL of gentamicin. Cell cultures were stimulated with MOG (20 μg/mL) or SAC (PANSORBIN®, Calbiochem®) (1/2500). Cytokine levels in the culture supernatants were evaluated 48 h later by enzyme-linked immunosorbent assay (ELISA) using IFN-γ, IL-5 and IL-10 BD OptEIA Sets (Becton Dickinson) and IL-17 (R&D Systems, Minneapolis, MN, USA). The assays were performed according to the manufacturer’s instruction.

### Proportion of CD4 + CD25 + Foxp3+ T Cells

Spleen cells were collected and the red blood cells were lysed with Hank’s buffer containing NH_4_Cl. Cells from the spleen and CNS were adjusted to 2.5 × 10^6^ cells/100 μL. These cells were then incubated with 0.5 μg of fluorescein isothyocianate (FITC) anti-mouse CD4 (clone GK1.5) and 0.25 μg of allophycocyanin (APC) anti-mouse CD25 (clone PC61.5) for 20 min at room temperature. Staining for Foxp3 was then performed using the phycoerythrin (PE) anti-mouse/rat Foxp3 Staining Set (eBioscience, San Diego, CA, USA) according to the manufacturer’s instructions. After incubation, the cells were fixed in 1% paraformaldehyde.The cells were analyzed by flow cytometry using the FACSCalibur (Becton Dickinson, San Jose, CA, USA) and BD CellQuest Pro software (Becton Dickinson, San Jose, CA, USA).

### Evaluation of inflammatory infiltrates in the CNS

The brain and lumbar spinal cord were obtained 30 days after EAE induction from infected and not infected animals. After fixation in 10% formaldehyde, the tissue samples were dehydrated in a graded ethanol series embedded in a 100% paraffin block. Five micron-thick sections were mounted onto glass slides, stained with hematoxylin and eosin and were analyzed using a Nikon microscope. Histopathologiocal analysis was also performed on brain samples from infected mice that were not subsequently immunized with MOG 15 days after infection.

### Statistical analysis

Data were expressed as the mean ± SE. Comparisons between groups were made using one-way ANOVA with post-hoc Holm-Sidak methods for parameters with normal distribution and using Kruskal-Wallis post-hoc Dunn’s method for parameters with non-normal distribution. The disease incidence was evaluated using Chi-square test (p = 0.051). The significance level was p < 0.05. Statistical analysis was achieved using SigmaStat for Windows v. 3.5 (Systat Software Inc.).

## Competing interests

The authors declare that they have no competing interests.

## Authors’ contributions

TGDF and AS are the main investigators in this study. FCM, SFGZP, LLWI, LCR and PMC largely contributed with the immunological experiments. CM and MRVI performed cytometric analysis. MLRSC provided critical input. All authors read and approved the final manuscript.
